# Cathepsin-D, a Key Protease in Breast Cancer, Is Up-Regulated in Obese Mouse and Human Adipose Tissue, and Controls Adipogenesis

**DOI:** 10.1371/journal.pone.0016452

**Published:** 2011-02-02

**Authors:** Olivier Masson, Christine Prébois, Danielle Derocq, Aline Meulle, Cédric Dray, Danielle Daviaud, Didier Quilliot, Philippe Valet, Catherine Muller, Emmanuelle Liaudet-Coopman

**Affiliations:** 1 IRCM, Institut de Recherche en Cancérologie de Montpellier, Montpellier, France; 2 INSERM, U896, Montpellier, France; 3 Université Montpellier 1, Montpellier, France; 4 CRLC Val d'Aurelle Paul Lamarque, Montpellier, France; 5 Université de Toulouse, UPS, Institut de Médecine Moléculaire de Rangueil, Toulouse, France; 6 Institute of Pharmacology and Structural Biology CNRS UMR 5089, Toulouse, France; 7 Université de Toulouse, Toulouse, France; 8 INSERM, U858, Toulouse, France; 9 Service de diabétologie, Maladies métaboliques et nutrition, CHU de Nancy, Nancy, France; Institut de Génomique Fonctionnelle de Lyon, France

## Abstract

The aspartic protease cathepsin-D (cath-D) is overexpressed by human epithelial breast cancer cells and is closely correlated with poor prognosis in breast cancer. The adipocyte is one of the most prominent cell types in the tumor-microenvironment of breast cancer, and clinical studies have shown that obesity increases the incidence of breast cancer. Here, we provide the first evidence that cath-D expression is up-regulated in adipose tissue from obese human beings, as well as in adipocytes from the obese C57BI6/J mouse. Cath-D expression is also increased during human and mouse adipocyte differentiation. We show that cath-D silencing in 3T3-F442A murine preadipocytes leads to lipid-depleted cells after adipogenesis induction, and inhibits of the expression of PPARγ, HSL and aP2 adipocyte differentiation markers. Altogether, our findings demonstrate the key role of cath-D in the control of adipogenesis, and suggest that cath-D may be a novel target in obesity.

## Introduction

The consumption of foods containing high levels of fat and carbohydrates is a major cause of obesity, resulting in the formation of excessive white adipose tissue. This increase in adipose tissue mass results from a combination of hypertrophy of existing adipocytes (hypertrophic adipocytes), and adipogenic differentiation of precursor cells (adipocyte hyperplasia). Recently, clinical studies have shown that obesity is a major risk factor for cancer [1], [2], [3]. The presence of large amounts of adipose tissues has been associated with poor prognosis for breast cancer in obese postmenauposal women [4].

Interestingly, proteases have also been recently shown to affect the biology of the adipocyte. The metalloproteinases [5], [6] and the cysteine cathepsins -K, -S and -L [7], [8], [9], [10] stimulate adipogenesis, and are up-regulated in obesity. In contrast, stromelysin 3 inhibits adipogenesis and induces de-differentiation of adipocytes, generating a population of fibroblast-like cells that support the desmoplastic reaction [11]. The aspartic protease cathepsin D (cath-D), a marker of poor prognosis in breast cancer [12], [13], [14], [15], [16], is overexpressed and secreted at high levels by human epithelial breast cancer cells [17], [18], [19], [20], [21], [22], [23]. Cath-D stimulates cancer cell proliferation, fibroblast outgrowth, angiogenesis and metastasis [24], [25], [26], [27], [28], [29], [30], [31], [32], [33], [34]. Interestingly, we recently published that the novel cath-D receptor, LRP1 (low-density lipoprotein receptor-related protein 1) [35], controls adipogenesis and is up-regulated in human and mouse obese adipose tissue [36].

Here, we investigated the expression of cath-D in adipocytes from obese subjects, and its role in the control of adipogenesis. We show, for the first time, that cath-D expression is up-regulated in mouse and human obese adipose tissues, as well as during mouse and human adipogenesis. We also demonstrate that cath-D silencing inhibits the adipogenic process, indicating the crucial positive role of cath-D in adipogenesis.

## Results

### Cath-D expression is up-regulated in human and mouse obese adipose tissue

Because of the recently established relationship between obesity and cancer incidence [1], [2], [3], and of the demonstrated role of cath-D in both cancer cells and stromal cells [21], we investigated cath-D expression in human and mouse adipose tissues.

**Figure 1:**
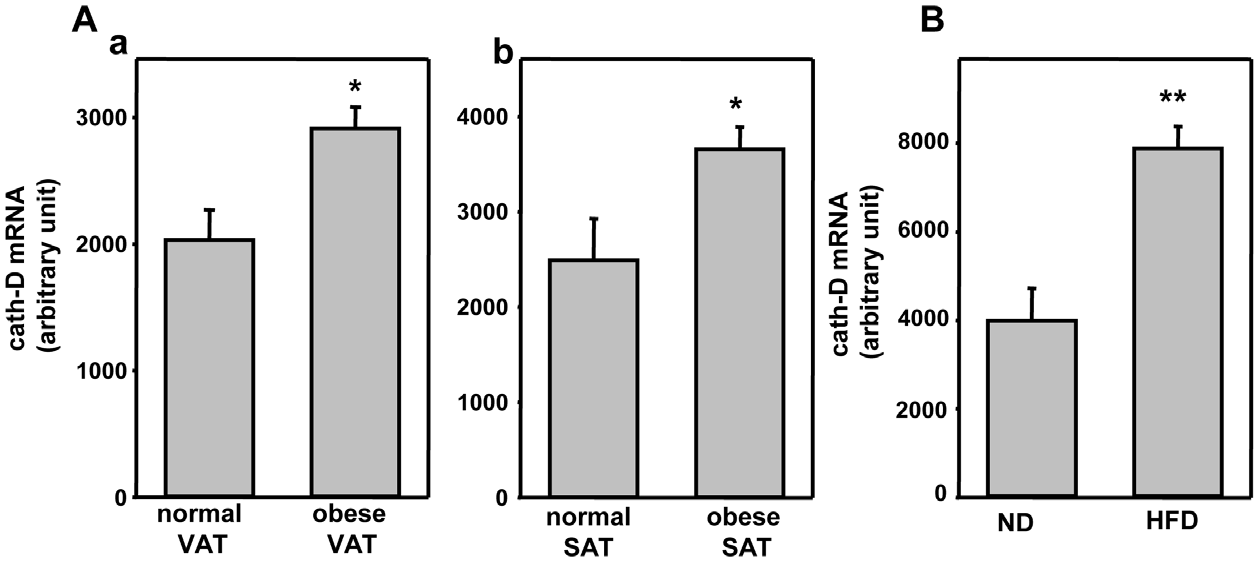
Cath-D expression is up-regulated in adipose tissues from obese human beings and mice. **(A) Cath-D expression in adipose tissue from lean and obese human subjects.** The cath-D mRNA level was quantified in samples of human intra-abdominal visceral adipose tissue (VAT) (panel a) and of human subcutaneous adipose tissue (SAT) (panel b) obtained from 27 morbidly (grade-III) obese patients (44.5+/−1.8 year old, BMI: 47.6+/−1.3 kg/m^2^) before they underwent bariatric surgery, and from 9 control patients about to undergo cosmetic abdominal lipectomy (42.7+/−4.5 year old, BMI: 23.1+/−3.3 kg/m^2^). Results are expressed as means +/− SEM, *P<0.01 *versus* controls (normal VAT or normal SAT). **(B) Cath-D expression in adipocytes from obese mice.** The cath-D mRNA level was quantified in adipocytes isolated from intra-abdominal adipose tissues from 30-week-old, overweight C57Bl6/J mice fed a high-fat diet (HFD), and from C57Bl6/J control mice fed on normal diet (ND). Results are means +/− SEM from 5 mice for ND group and 4 mice for HFD group. *P<0.005 *versus* ND controls.

Cath-D mRNA expression was investigated in intra-abdominal visceral adipose tissue (VAT) from lean and obese human subjects (Figure 1A, panel a). Interestingly, cath-D mRNA was significantly increased in the obese human visceral adipose tissue (Figure 1A, panel a). This differential expression of cath-D was also observed in subcutaneous adipose tissue (SAT) from lean and obese human subjects (Figure 1A, panel b).

In order to find out whether this up-regulation of cath-D was a general characteristic of adipocytes from obese subjects, we next analysed cath-D mRNA levels in adipocytes isolated from C57BI6/J mice fed either a High Fat Diet (HFD) or a Normal Diet (ND) (Figure 1B). HFD-fed C57BI6/J mice exhibited significantly higher body mass (47.6±1.4 g) than their control littermates (31.1±1.2 g). Cath-D expression was significantly greater in adipocytes from HFD obese mice than in those from ND control mice (Figure 1B). Overall, our results indicate that cath-D expression is up-regulated in adipose tissues of obese human beings and mice.

### Cath-D expression is increased in adipocytes during adipogenesis in mouse and human

Since we were not aware of any report establishing that cath-D protein is expressed in adipocyte cells, we analysed cath-D expression in well-established mouse adipocyte cell lines (3T3-F442A and 3T3-L1), and compared it to that in mouse fibroblasts (NIH-3T3). Cath-D is synthesized as a 52-kDa precursor that is rapidly converted in endosomes as an active 48-kDa single-chain intermediate, and then in the lysosomes into the fully active mature protease, composed of a 34 kDa heavy chain and a 14 kDa light chain. As Figure S1 shows, mouse cells mainly expressed the intermediate 48 kDa cath-D form, and only to a lesser extent the mature 34+14 kDa cath-D double chain, as previously described [37]. Interestingly, cath-D expression was increased in mature adipocytes in both the 3T3-F442A and 3T3-L1 cell lines (Figure S1).

**Figure 2:**
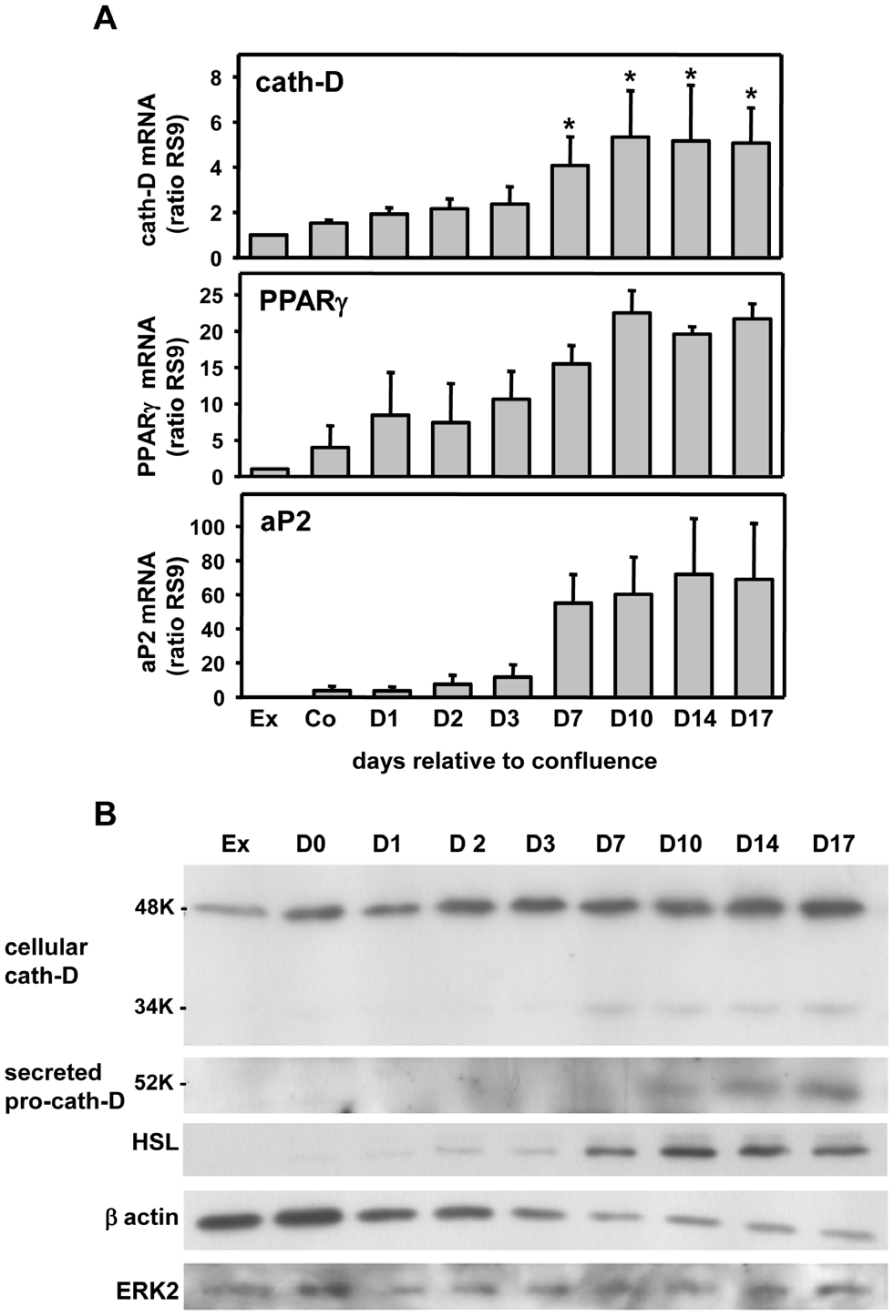
Cath-D expression increases during 3T3-F442A adipogenesis. **(A) Cath-D mRNA expression during adipogenesis.** RNA expression of cath-D, PPARγ and aP2 were analysed in exponentially growing 3T3-F442A preadipocytes (Ex), in 3T3-F442A cells grown to confluence (D0), and after culturing for the indicated days in adipogenic differentiation medium by real-time quantitative RT-PCR. Mean ± SD values from 4 independent experiments is shown. P<0.05 *versus* confluent adipocytes. **(B) Cath-D protein expression during adipogenesis.** The expression of the proteins cath-D, HSL, β-actin and ERK2 was analysed by immunoblotting in exponentially-growing 3T3-F442A preadipocytes (Ex), in 3T3-F442A adipocytes grown to confluence (D0), and the times indicated after induction of the differentiation process. Pro-cath-D secreted for 24h was analyzed during differentiation. ERK2 was used as a loading control. Similar results were observed in two independent experiments.

**Figure 3:**
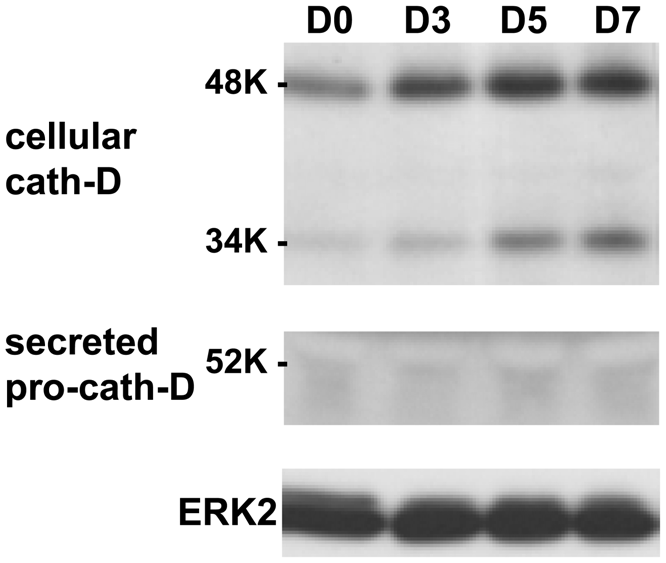
Cath-D expression increases during 3T3-L1 adipogenesis. Protein expression of cath-D was analysed by immunoblotting in 3T3-L1 adipocytes grown to confluence (D0), and after the differentiation process had been induced for the indicated days. The pro-cath-D secreted over 48h was monitored during differentiation. ERK2 was used as loading control.

**Figure 4:**
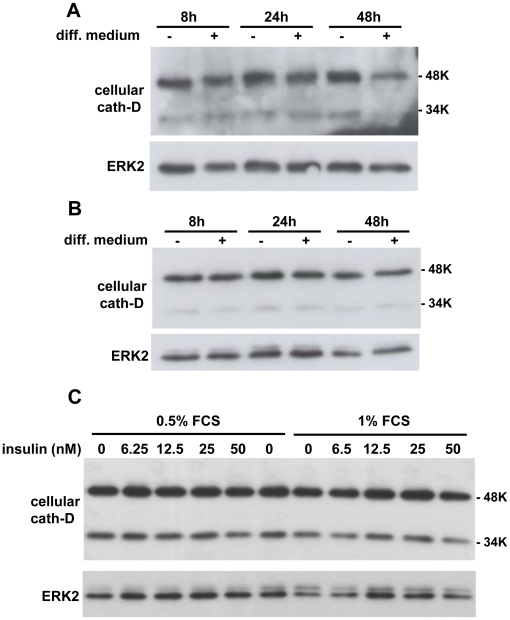
Effect of insulin on cath-D expression in NIH-3T3 fibroblasts and 3T3-F442A adipocytes. **(A) Cath-D expression in parental NIH-3T3 fibroblasts in the presence or the absence of the adipogenic differentiation medium.** Parental NIH-3T3 fibroblasts were grown or not for 8h, 24h and 48h in adipogenic differentiation medium (diff. medium). Cath-D protein expression was analysed by immunoblotting, and ERK2 was used as loading control. **(B) Cath-D expression in mature adipocytes in the presence or the absence of the adipogenic differentiation medium.** 3T3-F442A adipocytes differentiated for 10 days were grown or not for 8h, 24h and 48h in adipogenic differentiation medium (diff. medium). Cath-D protein expression was analysed by immunoblotting, and ERK2 was used as loading control. **(C) Effect of increasing doses of insulin on cath-D expression in adipocytes.** 3T3-F442A adipocytes differentiated for 10 days were treated with increasing doses of insulin (6.25 nM to 50 nM) in 0.5% or 1% FCS for 24h. Cath-D protein expression was analysed by immunoblotting. ERK2 served as loading control.

Obesity is characterized by the increased intracellular accumulation of lipids, a characteristic of adipocyte differentiation that is significantly correlated with adipocyte differentiation. Since cath-D expression was up-regulated in obese tissues, we next investigated whether cath-D plays any role in adipogenesis. We first investigated the regulation of cath-D expression during the course of differentiation of the 3T3-F442A preadipocyte cell line, a valuable model of adipogenesis [38] (Figure 2). Interestingly, cath-D mRNA (Figure 2A) and protein (Figure 2B) expression was progressively up-regulated during adipogenesis. Since the overexpression of cath-D leads to the hypersecretion of the 52 kDa pro-cath-D into the extracellular environment [19], we then investigated whether adipocytes secrete 52 kDa pro-cath-D. Secreted 52 kDa pro-cath-D was only detected in fully-differentiated adipocytes from day 10 of differentiation (Figure 2B). To validate our experimental conditions, we studied in parallel the expression of PPARγ (Figure 2A), HSL (Figure 2B) and aP2 (Figure 2A) adipocyte markers of differentiation. As expected, the levels of these markers increased progressively during the acquisition of the adipocyte phenotype (Figure 2A–B). In addition, the amount of cytoskeletal β-actin protein decreased during adipocyte differentiation, reflecting the change in cellular morphology (Figure 2B), as previously described [39]. Interestingly, a similar up-regulation of cath-D protein expression was observed during 3T3-L1 adipocyte differentiation (Figure 3). It was previously shown that cath-D expression was stimulated by insulin in epithelial breast cancer cells [40], [41]. To ensure that the insulin treatment was not involved in the effect observed, we used parental NIH-3T3 fibroblasts and fully-differentiated 3T3-F442A adipocytes and. As shown in Figure 4, the levels of cath-D protein remained unaffected in the adipogenic differentiation medium both in NIH-3T3 fibroblasts (Figure 4A) and in mature 3T3-F442A adipocytes (Figure 4B). We also confirmed that cath-D protein expression was not altered in 3T3-F442A adipocytes by insulin in dose-response experiments at low serum concentrations (Figure 4C). Altogether, these results strongly suggest that cath-D up-regulation is induced by the differentiation process *per se* and not by insulin.

**Figure 5:**
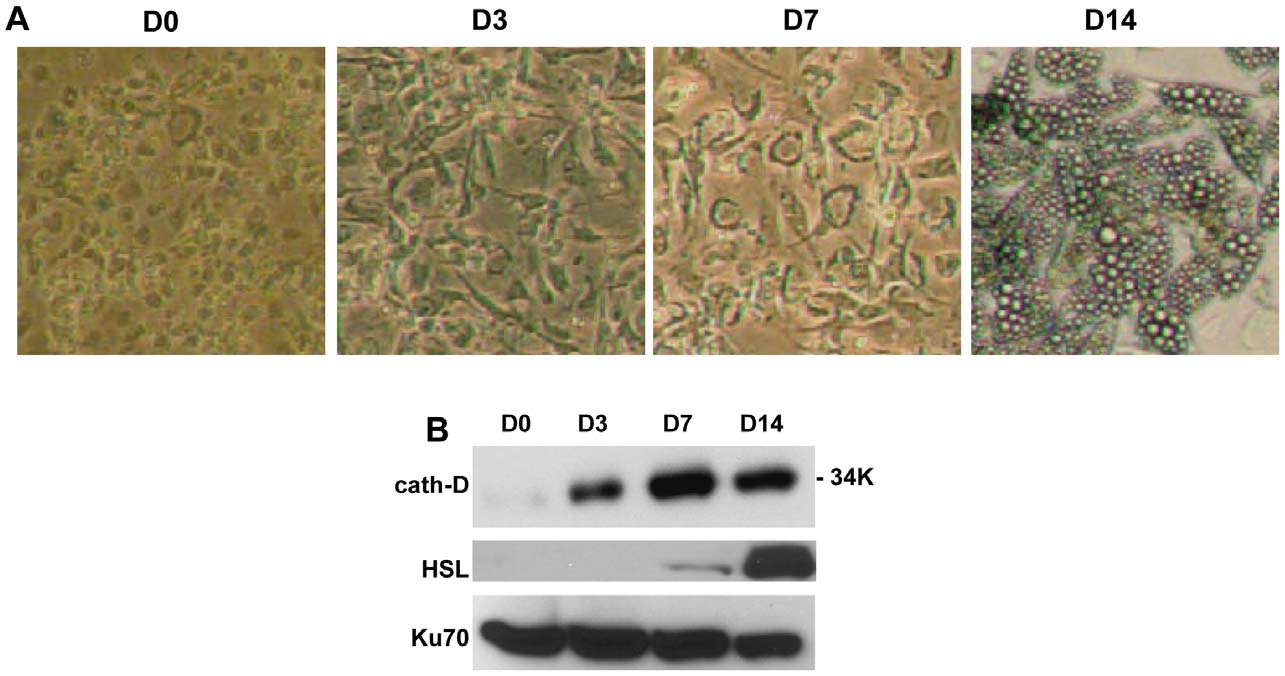
Expression of cath-D during adipogenesis in human. **(A) Micrographs of human adipocytes.** Human preadipocytes isolated from subcutaneous adipose tissue digested with collagenase and separated from the stromal vascular fraction were grown for 0, 3, 7 and 14 days in the presence of the adipogenic medium as illustrated in the micrographs. A representative experiment is shown. **(B) Cath-D expression during adipogenesis.** Protein expression of cath-D and HSL was analysed by immunoblotting after the indicated time following induction of the differentiation process (panel a). Ku70 was used as loading control, as previously described [52]. One representative experiment out of 2 is shown.

Although 3T3-F442A and 3T3-L1 cells are valuable experimental models, these preadipocytes have some distinct attributes compared with human cells in primary culture beyond the obvious species differences. We therefore next investigated the regulation of cath-D expression during adipogenesis in human preadipocytes purified from abdominal subcutaneous adipose tissue [42] (Figure 5). These primary human cells were differentiated in an efficient manner since about 75% of preadipocytes were converted into the adipocyte phenotype (Figure 5A). As observed for the mouse adipocyte, the expression of cath-D protein was also increased in the human differentiated adipocyte (Figure 5B). Taken together, these findings indicate that cath-D expression is up-regulated during the differentiation of both mouse and human preadipocytes.

### Silencing of cath-D expression inhibits adipogenesis

**Figure 6:**
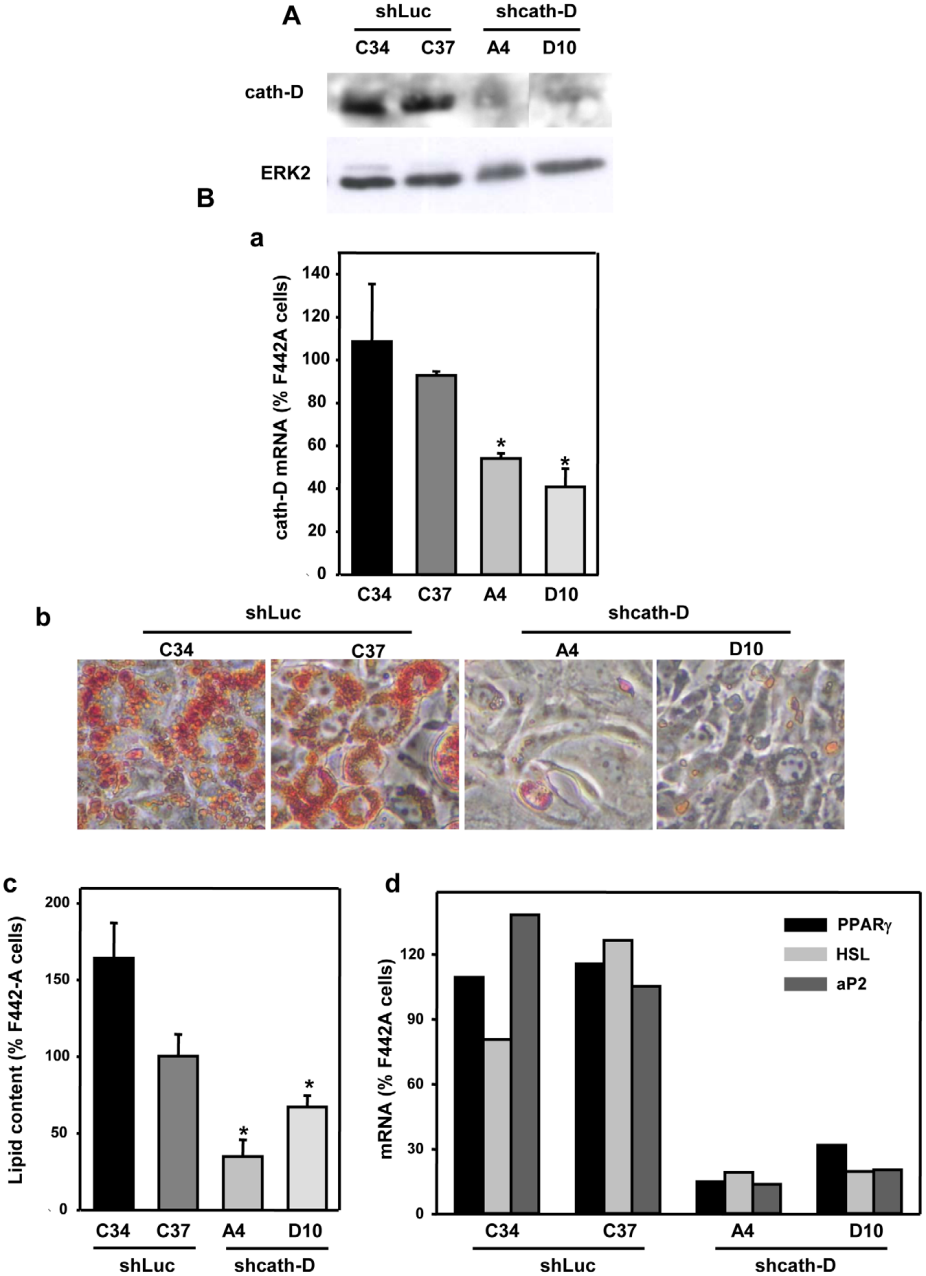
Silencing of cath-D in 3T3-F442A preadipocytes inhibits adipogenesis. **(A) Silencing of cath-D by shRNAs in 3T3-F442A preadipocytes.** 3T3-F442A preadipocytes were stably transfected with Luc shRNA (C34 and C37 clones), cath-D shRNA1 (D10 clone) and cath-D shRNA2 (A4 clone). Cath-D expression was monitored by immunoblotting in C34, C37, A4, and D10 clones. α-tubulin was used as a loading control. **(B) Extinction of cath-D expression in 3T3-F442A preadipocytes inhibits adipogenesis.** Luc shRNA (C34 and C37) and cath-D shRNA (A4 and D10) clones were maintained for 7 days in the adipogenic differentiation medium. Cath-D mRNA expression was quantified in C34, C37, A4 and D10 clones at day 7 of differentiation (panel a). Mean ± SD of 3 independent experiments is shown. *P<0.005 *versus* the C37 clone. Micrographs of Luc shRNA (C34 and C37) and cath-D shRNA (A4 and D10) clones were performed at day 7 of differentiation (panel b). One representative experiment out of 3 is shown. The lipid content was quantified at day 7 of differentiation in Luc shRNA (C34 and C37) and cath-D shRNA (A4 and D10) clones (panel c). Mean ± SD of triplicate of is shown. *P<0.025 *versus* the C37 clone. Expression of PPARγ, HSL and aP2 mRNA was analysed in Luc shRNA clones (C34 and C37) or cath-D shRNA clones (A4 and D10) after 7 days in the adipogenic differentiation medium (panel d). One representative experiment out of 2 is shown.

Since cath-D was up-regulated in tissues from obese individuals, and as its expression increased during the adipocytic process, we went on to investigate whether preadipocytes require cath-D expression to differentiate into mature adipocytes. Cath-D expression was stably silenced in 3T3-F442A preadipocytes with cath-D shRNA1 and shRNA2 generating the D10 and A4 clones, respectively (Figure 6A). Control C34 and C37 clones were obtained using Luc shRNA stably transfected into 3T3-F442A preadipocytes (Figure 6A).

To evaluate the consequences of cath-D silencing on adipocyte differentiation, we analysed the cellular lipid levels in adipocytes which had or had not been silenced for cath-D at day 7 of differentiation (Figure 6). Indeed, the most obvious feature of adipocytes is the fact that they synthesise and store triglycerides in lipid droplets and therefore the gradual development of lipid droplets is characteristic of adipocyte precursor cells undergoing adipogenic differentiation. Quantification of cath-D mRNA at day 7 of differentiation revealed a significant inhibition of cath-D expression by 56+/−2.4% and 60+/−8.5% in A4 and D10 clones, respectively (Figure 6B, panel a). As illustrated in Figure 6B (panel b), detection of neutral lipid by oil red O staining after 7 days of differentiation revealed that C34 and C37 clones accumulated numerous large lipid droplets and adopted the non-adherent, round morphology characteristic of mature 3T3-F442A adipocytes. In contrast, in A4 and D10 clones silenced for cath-D, the lipid droplets were markedly smaller and fewer in number (Figure 6B, panel b). A4 and D10 clones retained the morphology of adherent fibroblastic cells, suggesting that the preadipocyte differentiation process did not occur in the absence of cath-D (Figure 6B, panel b). Quantification of lipids demonstrated that silencing cath-D in A4 and D10 clones resulted in significantly lower lipid content at day 7 of differentiation than in the C34 and C37 control clones (Figure 6B, panel c). We finally analysed the expression of PPARγ, HSL and aP2 adipocyte differentiation markers in Luc and cath-D shRNA-transfected 3T3-F442A clones at day 7 of differentiation (Figure 6B, panel d). Cath-D silencing was associated with inhibition of PPARγ, HSL and aP2 mRNA levels (Figure 6B, panel d). Taken together, these findings indicate that cath-D silencing in preadipocytes inhibits adipogenesis, leading to lipid-depleted cells.

## Discussion

Our results demonstrate, for the first time, that cath-D expression is up-regulated in abdominal visceral and subcutaneous tissues from obese human subjects. A previous study observed cath-D mRNA expression in abdominal subcutaneous adipose tissue [43]. This up-regulation of cath-D expression in the VAT and SAT of overweight/obese patients is consistent with our data from obese mice. Obesity is characterized by increased intracellular accumulation of lipids, which is significantly correlated with adipocyte differentiation. Terminally differentiated adipocytes cannot divide, which means that any increase in the number of adipocytes in the body must result from the differentiation of preadipocytes, which act as a renewable source of adipocytes.

Our data show that cath-D expression increases gradually as 3T3-F442A and 3T3-L1 cells differentiate into mature adipocytes. Interestingly, our findings also reveal that fully-differentiated 3T3-F442A adipocytes secrete pro-cath-D, suggesting a potential new function as an adipokine. A recent study analysing the secretome of adipocytes reported that cath-D is a secretory protein induced by insulin [44]. Most functional studies of adipocyte differentiation and function have been performed using the murine adipogenic 3T3-L1 and 3T3-F442A cell lines or genetically-modified mice. However, there are fundamental differences in lipoprotein metabolism between mice and human beings [45]. This is why it was important to investigate the regulation of cath-D expression in human adipocytes. We observed the up-regulation of cath-D during adipogenesis in a primary culture of preadipocytes isolated from human sub-cutaneous adipose tissue. Interestingly, cath-D expression has also been shown to be markedly increased during the differentiation process in primary cultures of brown adipocytes [46].

Our report highlights that cath-D silencing by shRNAs in 3T3-F442A preadipocytes leads to lipid-depleted cells, and to a reduction of the expression of PPARγ, HSL and aP2 adipocyte markers of differentiation, indicating the essential role of cath-D in the differentiation process of preadipocytes. Given that cath-D protein is required for adipocyte differentiation and as it is abundantly expressed in fully-differentiated adipocytes, we propose that cath-D may be involved in the onset of obesity. Moreover, murine cath-D deficiency led to the accumulation of cholesteryl esters in the brain, suggesting that cath-D may play a key role in lipid metabolism [47].

Taken together, our findings reveal that cath-D is up-regulated in tissues from obese humans and mice, and that it is a novel key regulator of adipogenesis. We propose that targeting cath-D in obesity could reduce the number of hypertrophic adipocytes and decrease adipocyte hyperplasia. In the future, we intend to investigate the mechanism(s) by which cath-D controls adipogenesis. Since experimental studies have demonstrated that adipocytes play a supportive role in breast growth [48], [49], [50], and as clinical studies have shown that obesity increases the incidence of breast cancer [1], [2], [3], we will analyse the role of cath-D up-regulation in obese adipocytes with regard to cancer.

## Materials and Methods

### Ethics Statement

All subjects gave their informed written consent to participate to the study.

Our protocol is using adipose tissues from plastic surgery considered as “waste”, and we thus do not need a specific agreement. However the study was performed accordingly to the declaration of Helsinki as revised in 2000 (http://www.wma.net/e/policy/b3.htm) and approved by the Ethical commitee of the Rangueil hospital (Toulouse, France) with permit number 07/858/03/07. Mice were handled in accordance with National Institute of Medical Research (INSERM) principles and guidelines. C57Bl6/J female mice were obtained from Charles River laboratory (l'Arbresle, France). Philippe Valet, with the license number # 31–147 given by the French government, performed the experiments on the animals. This study was approved by the ethics committee of Midi-Pyrénées (France) with the permit number MP/01/01/01/2010.

### Cells and cell culture

Cell lines were cultured in DMEM (Invitrogen) supplemented with 10% fetal calf serum (FCS). Differentiation was induced by incubating confluent 3T3-F442A cells in differentiation medium (DMEM supplemented with 10% FCS and 50 nM insulin) as described [45]. Differentiation was induced by incubating 3T3-L1 confluent cells in differentiation medium (DMEM supplemented with 10% FCS and 10 µg/ml insulin, 250 µM isobutylmethylxanthine, 1 µM rosiglitazone, 1 µM dexamethasone).

### RNA extraction and analysis

Total RNA was extracted using the Reasy minikit (QIAGEN Sciences, Maryland) according to the manufacturer's instructions. Reverse transcription of total RNA was performed at 37°C using Moloney murine leukemia virus reverse transcriptase enzyme (Invitrogen, Carlsbad, CA) and random hexanucleotide primers (Promega, Madison, WI). Quantitative PCR was carried out by real-time PCR using a LightCycler and the DNA double-strand-specific SYBR green I dye for detection (Roche, Basel, Switzerland). Results for mouse RNA were normalized to RS9 levels. For human RNA, analysis of the 18 S ribosomal RNA was performed in parallel using the ribosomal RNA control Taqman Assay Kit (Applied Biosystem) to normalize gene expression.

The sequences of the primers were:

mouse RS9 (sens 5′CGGCCCGGGAGCTGTTGACG3′, reverse 5′CTGCTTGCGGACCCTAATGTGACG3′),

mouse aP2 (sens 5′AACACCGAGATTTCCTTCAA3′, reverse 5′AGTCACGCCTTTCATAACACA3′),

mouse cath-D (sens 5′TTCGTCCTCCTTCGCGATT3′; reverse

5′TCCGTCATAGTCCGACGGATA3′)

mouse HSL (sens 5′CTGAAGGCTCTGAGTTGGTCAA3′, reverse 5′GGCTTACTGGGCACAGATACCT3′),

mouse PPARγ (sens 5′ AGGCCGAGAAGGAGAAGCTGTTG3′, reverse 5′TGGCCACCTCTTTGCTCTGCTC3′),

human cath-D (5′TTGCTGTTTTGTTCTGTGGTTTTC′, reverse 5′CAGACAGGCAGGCAGCATT3′).

### Stable transfection of shRNAs in 3T3-F442A cells

3T3-F442A cells were transfected with 1 µg of shLuc, anti-cath-D shRNA1 or shRNA2 expression vectors (Invivogen) using Nucleofector Technology (Amaxa biosystems) according to the manufacturer's instructions and 40 clones resistant to Blasticidin (4 µg/ml) were isolated. Clone D10 transfected with anti-cath-D shRNA1 and clone A4 transfected with anti-cath-D shRNA2 were the best clones selected for cath-D silencing.

sh1 5′GGTTCCATGTAAGTCTGACCATCAAGAGTGGTCAGACTTACATGGACCC3′

sh2 5′GACCAGTCAAAGGCAAGAGGTTCAAGAGACCTCTTGCCTTTGACTGGTC3′

### Oil Red O staining

Adipocytes were washed with phosphate-buffered saline (pH 7.4) and then fixed with Antigenfix (Diapath, Italy). Cells were stained with Oil Red O dye (saturated Oil Red O dye in six parts of isopropanol and four parts of water), an indicator of cell lipid content, and then exhaustively rinsed with water. Spectrophotometric quantification of the stain was performed by dissolving the stained oil droplets in the cell in isopropanol and measuring their absorbance at 540 nm.

### Human samples

Human adipose tissue was collected according to the guidelines of the Ethical Committee of Toulouse-Rangueil and Nancy J. d'Arc Hospitals and with the full ethical approval of the Ethical Committee of Toulouse-Rangueil and Nancy J. d'Arc Hospitals. All subjects gave their informed written consent to participate in the study. Human abdominal visceral (VAT) adipose tissue and human subcutaneous adipose tissue (SAT) samples were obtained from 9 healthy volunteer patients (42.7+/−4.5 yr old, BMI: 23.1+/−3.3 kg/m^2^) undergoing cosmetic abdominal lipectomy. No clinical data from these patients were available. Human VAT adipose tissue samples were obtained from 27 morbidly (grade-III) obese subjects (44.5+/−1.8 yr old, BMI: 47.6+/−1.3 kg/m^2^) before they underwent bariatric surgery.

All subjects were drug-free, and were not suffering from any disorder other than obesity. Tissue samples were immediately frozen in liquid nitrogen, and stored at −80°C. Total RNAs were extracted from isolated adipocytes, and their cath-D expression analysed by RT-PCR.

For *in vitro* differentiation, human preadipocytes were isolated from human subcutaneous adipose tissue obtained from patients undergoing abdominal lipectomy in the plastic surgery department of Rangueil Hospital (Toulouse, France) with the approval of the locals Ethic Committee. All subjects gave their informed written consent to participate in the study. Adipose tissue fragments were immediately used for collagenase digestion, as previously described [42]. The digestate was centrifuged to separate the adipocytes from the stroma-vascular fraction, which contained preadipocytes (pellet). Cells isolated from the SVF fraction were induced to differentiate into adipocytes as previously described [51]. Briefly, confluent cells (day 0) were induced to differentiate in DMEM/Ham's F12 (1∶1) medium containing 0.01 mg/ml transferrin, 100 nM cortisol, 0.2 nM triiodothyronine, and 20 nM insulin. To trigger differentiation, 25 nM dexamethasone, 500 mM IBMX and 2 mM rosiglitazone were included from day 0 to day 4. Intracellular accumulation of lipid droplets became clearly evident at day 10 [42].

### Mice

Mice were handled in accordance with National Institute of Medical Research (INSERM) principles and guidelines. C57Bl6/J female mice were obtained from Charles River laboratory (l'Arbresle, France). Mice were housed conventionally in an animal room at constant temperature (20–22°C) and humidity (50–60%), and with a 12 h light–dark cycle. All the mice had free access to food and water throughout the experiment. The C57Bl6/J mice were assigned to a normal diet (ND) or high-fat diet (HFD) (SAFE, France). The energy contents of the diets were as follows: 20% protein, 70% carbohydrate, and 10% fat for the ND; and 20% protein, 35% carbohydrate, and 45% fat for the HFD. The main source of fat in the HFD was lard (20 g/100g of food). C57Bl6/J mice (initially 10 weeks old) were fed an ND or HFD for 20 weeks. A 20 weeks period of HFD is used to mimic the slow development of obesity in humans associated with the settlement of insulin resistance and low grade inflammation. All the mice were sacrificed at 30 weeks of age.

### Isolation of adipocytes from mouse adipose tissue

Mouse intra-abdominal adipose tissues were dissected immediately after sacrifice, minced in 5 ml of Dulbecco's modified Eagle's medium (DMEM; Life Technologies, Inc., Invitrogen, Paisley, UK) supplemented with 1 mg/ml collagenase (SIGMA) and 1% BSA for 30 min at 37°C while shaking. Digestion was followed by filtration through a 150 µm screen, and the floating adipocytes were separated from the medium containing the stroma-vascular fraction (SVF). Adipocytes were washed twice in DMEM, and then processed for RNA extraction using the RNeasy mini kit (Qiagen, Germany).

### Immunoblots

Cells were lysed in lysis buffer (50 mM HEPES pH 7.5, 150 mM NaCl, 10% glycerol, 1% Triton X100, 1.5 mM MgCl_2_, 1 mM EGTA, 100 mM NaF, 10 mM NaPPI, 500 µm Na-Vanadate, 1 mM PMSF, 10 µM Aprotinine, and a protease inhibitor cocktail). After shaking gently for 20 min at 4°C, cell extracts were obtained by centrifugation in a microfuge at 13,000 rpm for 15 min at 4°C. Equal amounts of protein (100 µg) from cell extracts, quantitated by the Bradford assay, were separated on a 7% gel by SDS-PAGE. Proteins were electro-transferred to a PVDF membrane, and then incubated with 1 µg/ml anti-mouse cath-D (Santa Cruz Biotechnology), 1 µg/ml anti-human cath-D (BD Biosciences), 1 µg/ml anti-α tubulin (Lab Vision Corporation), 1 µg/ml anti-β actin (Sigma), 1 µg/ml ERK2 (Santa Cruz Biotechnology), or 0.4 µg/ml anti-HSL (Santa Cruz Biotechnology). Proteins were visualized with horseradish peroxidase-conjugated sheep anti-mouse immunoglobulin (ECL Amersham) or horseradish peroxidase-conjugated rabbit anti-goat immunoglobulin (ECL Amersham) followed by the Renaissance chemiluminescence system (Perkin Life Sciences).

### Statistical analysis

Results are expressed as means ± SEM. Statistical differences between two groups were evaluated using Student's *t* tests. The level of significance was set at *P*<0.05.

## Supporting Information

Figure S1 **Cath-D expression in preadipocytes, adipocytes, and fibroblasts.** Cath-D expression was analysed by immunoblotting confluent preadipocytes (D0), and adipocytes differentiated for 7 days (D7) from the 3T3-F442A and 3T3-L1 preadipocytic cell lines, and NIH-3T3 mouse fibroblasts. α-tubulin was used as a loading control.
(TIF)
