# Supplementary figures and images for: Cathepsin-D, a Key Protease in Breast Cancer, Is Up-Regulated in Obese Mouse and Human Adipose Tissue, and Controls Adipogenesis
Source: PLoS One. 2011 Feb 2;6(2):e16452. doi: 10.1371/journal.pone.0016452 (PMC3032791; doi:10.1371/journal.pone.0016452)

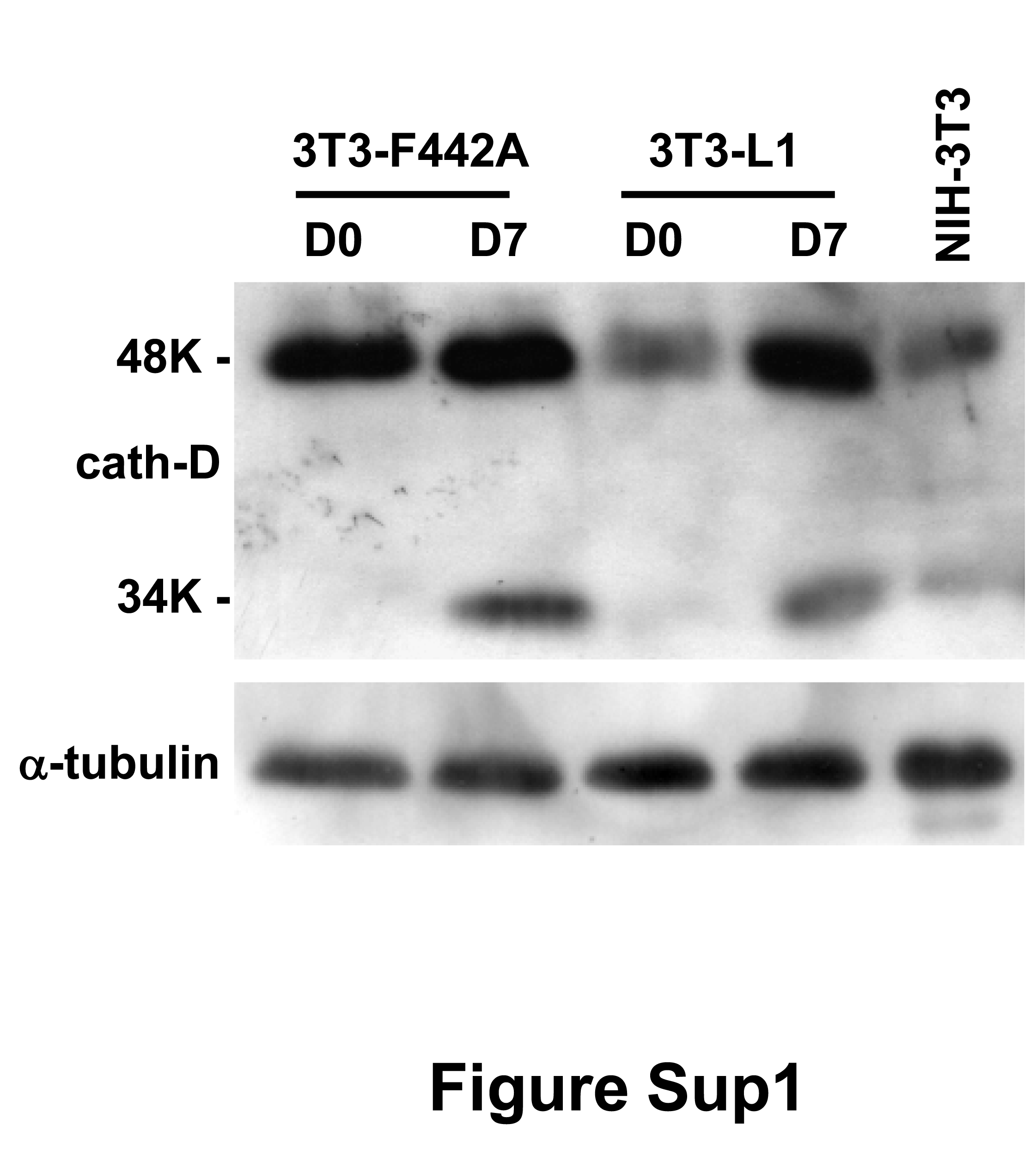

Supplement: Figure S1 — Cath-D expression in preadipocytes, adipocytes, and fibroblasts. Cath-D expression was analysed by immunoblotting confluent preadipocytes (D0), and adipocytes differentiated for 7 days (D7) from the 3T3-F442A and 3T3-L1 preadipocytic cell lines, and NIH-3T3 mouse fibroblasts. α-tubulin was used as a loading control. (TIF) [file pone.0016452.s001.tif]
